# Measures for Identifying Malnutrition in Geriatric Rehabilitation: A Scoping Review

**DOI:** 10.3390/nu16020223

**Published:** 2024-01-10

**Authors:** Shinta Nishioka, Yoji Kokura, Ryo Momosaki, Yutaka Taketani

**Affiliations:** 1 Department of Clinical Nutrition and Food Service, Nagasaki Rehabilitation Hospital, 4-11, Gin-yamachi, Nagasaki 850-0854, Japan; 2 Department of Clinical Nutrition and Food Management, Institute of Biomedical Sciences, Tokushima University Graduate School, 3-18-15, Kuramoto-cho, Tokushima 770-8503, Japan; taketani@tokushima-u.ac.jp; 3 Department of Nutrition Management, Keiju Hatogaoka Integrated Facility for Medical and Long-Term Care, 15-39-8, Mugigaura, Anamizu, Hosu-gun 927-0023, Japan; yojikokura@hotmail.com; 4 Department of Rehabilitation Medicine, Mie University Graduate School of Medicine, 2-174, Edobashi, Tsu 514-8507, Mie, Japan; momosakiryo@gmail.com

**Keywords:** disability, diagnostic criteria for malnutrition, geriatric rehabilitation, malnutrition, nutritional assessment

## Abstract

Malnutrition is a common condition in geriatric rehabilitation settings; however, the accuracy and predictive validity of the measures to identify malnutrition have not been established. The current scoping review followed the Joanna Briggs Institute’s evidence synthesis manual and the Preferred Reporting Items for Systematic Reviews and Meta-Analysis Extension for Scoping Reviews checklist. Literature published through September 2023 was searched using MEDLINE and CINAHL. The inclusion criteria selected studies reporting malnutrition measures, which include static body weight and weight loss. Identified tools were classified as nutritional screening tools, nutritional assessment tools, or diagnostic criteria. The domains of each tool/criterion and their accuracy and predictive validity were extracted. Fifty-six articles fulfilled the inclusion criteria, and six nutritional screening tools, three nutritional assessment tools, and three diagnostic criteria for malnutrition were identified. These measures consisted of various phenotypes, e.g., weight loss, causes such as inflammation/disease, and risk factors of malnutrition, e.g., functional impairment. The predictive validity of nutritional screening tools (*n* = 6) and malnutrition diagnostic criteria (*n* = 5) were inconsistently reported, whereas those for nutritional assessment tools were scarce (*n* = 1). These findings highlight the need to distinguish the functional impairment of nutritional origin from that of non-nutritional origin in nutritional assessment procedures, and the need to study the accuracy and the predictive validity of these measures in geriatric rehabilitation patients.

## 1. Introduction

Malnutrition is a comprehensive term to describe under- and overnutrition, but it is primarily used for undernutrition [1,2]. It is recognized as a critical health issue in older adults worldwide due to its serious consequences, such as increased morbidity, healthcare costs, and mortality [1,2]. The prevalence of malnutrition varies, depending on the setting and measurements used, with 3.1% of these patients in community settings, 22% in hospitals, 17.5% in nursing homes, and 29.5% in geriatric rehabilitation centers [3].

Older patients in rehabilitation settings seem most vulnerable to malnutrition [4]. Growing attention is being paid to nutritional problems of rehabilitation patients, because of their association with a poorer recovery of functional capabilities and an increased risk of institutionalization and hospitalization [5,6,7,8]. Reduced physical function, e.g., dysphagia and an inability to eat, an inflammatory response to acute/chronic disease, socioeconomic status, and polypharmacy explain the high prevalence of malnutrition [5,9]. Moreover, malnutrition per se can exacerbate physical disabilities through muscle weakness and fatigue and may result in extended hospitalization. We refer to this vicious spiral as a “malnutrition–disability cycle” [9]. Thus, multidimensional rehabilitation teams should involve nutrition professionals, e.g., registered dietitians, to assess the nutritional statuses of geriatric rehabilitation patients and to provide nutritional care.

Nutritional assessment is crucial to identify the severity and etiology of malnutrition and to determine appropriate nutritional therapy. After nutritional screening, nutritional assessment is undertaken by nutrition professionals with validated tools, involving a variety of nutrition-related factors, e.g., food intake, weight loss, body weight, dietary pattern, disease burden, and muscle and fat accumulation. This process is performed using validated tools such as the Mini Nutritional Assessment (MNA) [10], the Subjective Global Assessment (SGA) [11], and the Patient-Generated SGA (PG-SGA) [12]. Furthermore, diagnostic criteria for malnutrition, such as those from the Global Leadership Initiative on Malnutrition (GLIM), are spreading worldwide.

However, the criterion validity of nutritional assessment tools and diagnostic criteria for malnutrition in geriatric rehabilitation settings have not been well investigated. Criterion validity indicates what the index test assesses, and is typically classified as concurrent or predictive validity [13]. Concurrent validity, i.e., accuracy, reflects the extent of agreement or association between scores obtained from a given test and the “gold standard” method. To date, few studies have examined the accuracy of measures for malnutrition in geriatric rehabilitation patients [13]. On the other hand, predictive validity refers to the ability of a given test to predict future outcomes. Like accuracy, the predictive validity of malnutrition measures for older rehabilitation patients has not been established. The reason is that most investigations have been derived from cross-sectional studies of poor quality that cannot draw firm conclusions regarding cause-and-effect relationships [9]. Moreover, the interpretation of predictive validity for functional outcomes is often difficult, as some nutritional assessment tools contain a physical function domain, resulting in the overestimation of the correlation between tools and outcomes [9]. These issues underscore the need to comprehensively evaluate malnutrition measures used for geriatric rehabilitation patients and to examine their criterion validity.

Therefore, this scoping review examined measures used to identify malnutrition in geriatric rehabilitation patients. It also investigated measures used to identify malnutrition and evaluated their accuracy and predictive validities.

## 2. Materials and Methods

The protocol of this scoping review was based on the framework proposed by Arksey and O’Malley [14] and advanced by Levac [15]. The Joanna Briggs Institute’s evidence synthesis manual [16] and the Preferred Reporting Items for Systematic Reviews and Meta-Analysis Extension for Scoping Reviews checklist were also utilized [17]. The review team included two independent researchers with expertise in clinical nutrition, two supervisors with nutritional science and rehabilitation medicine expertise, and a librarian. The principal investigator established the review protocol and then critically revised it, assisted by other researchers. The primary research questions were as follows:

- What are the characteristics of existing measures for identifying malnutrition in geriatric rehabilitation patients?
- What are the accuracy and predictive validities of these methods?

The inclusion criteria for the literature search implemented the “patient, concept and context” framework (Table 1). The current review focused on all nutritional assessment methods utilized for older adults in geriatric rehabilitation settings. The study design examined only cross-sectional studies, cohort studies, case–control studies, systematic reviews, meta-analyses, and intervention studies.

Scholarly articles that investigated older adults (mean age ≥ 65 years), performed nutritional assessments, and were published in English between 1 January 1980 and 31 May 2022 were included (Table S1). Publications were restricted to observational and intervention studies, systematic reviews, and meta-analyses. Nutritional assessments employed composite methods, developed by the Academy of Nutrition and Dietetics Evidence Analysis Center, including a static measure of body weight or body mass index (BMI), and changes in body weight and BMI [18]. Published papers involving older adults in acute care or community settings, applying nutritional assessment methods that did not fulfil the pre-established criteria, or written in languages other than English, were excluded.

A literature search was performed using MEDLINE and CINAHL on 3 July 2022. An additional search was conducted on 11 October 2023, to include recent literature as of 30 September 2023. Search terms were developed by the principal researcher and then refined by a librarian (Table S2). After excluding duplicate papers, two researchers independently screened the literature by titles and abstracts, using Rayyan [19]. Full-text screening for retrieved articles was subsequently performed to extract studies within the scope of this review. Discrepancies in judgements between the two researchers were resolved by discussion.

An information sheet was used to extract the following data: study populations, numbers and characteristics of participants, the study design, malnutrition prevalence (or its risk prevalence for nutritional screening tools), method and timing of nutritional assessments, and occupations of individuals implementing nutritional assessment. Information on domains of nutritional assessment measures and their accuracy and predictive validity were investigated. Indicators for accuracy included sensitivity and specificity.

The current review classified measures for identifying malnutrition into three categories: (1) nutritional screening tools, which comprise simple and readily available measurements, designed to screen individuals at risk of malnutrition [2,20]. (2) Nutritional assessment tools that involve more complex, multidimensional information related to malnutrition [1]. (3) Diagnostic criteria for malnutrition, intended to diagnose malnutrition using criteria such as the GLIM criteria [1], established by nutrition experts. Implementing this categorization was justified because the recommended process for evaluating nutritional status in older adults comprises nutritional screening, followed by detailed nutritional assessment, as well as a diagnosis of malnutrition [4]. Note that nutritional screening tools are not designed for the assessment of nutritional status. Similarly, the diagnostic criteria for malnutrition should be distinguished from nutritional assessments, as they aim to diagnose malnutrition but not evaluate nutritional status comprehensively [1]. Nonetheless, this review did not exclude studies that used nutritional screening tools and diagnostic criteria for malnutrition because these are often applied to present malnutrition in both clinical practice and research.

A mapping matrix illustrated what outcome measures were investigated in the literature by each nutritional assessment method. Assessing nutritional status before adverse events and addressing confounding effects are essential to examine the predictive validity of nutritional assessment methods [9]. Thus, cross-sectional studies and results without adjustment for confounding were excluded from the mapping matrix.

## 3. Results

**Figure 1:**
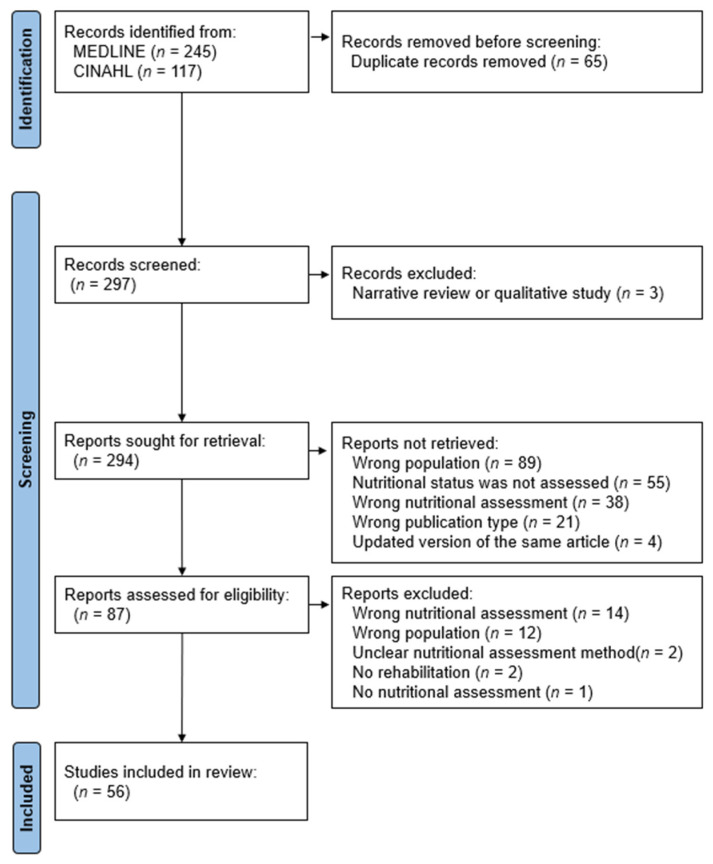
Flow chart of publication selection for this scoping review.

Electronic searches identified 362 articles. After excluding 65 duplicate records, 297 articles were screened and 294 studies were retrieved (Figure 1). Of these, 87 articles were eligible for full-text review. After excluding 31 more publications, 56 were included in this scoping review [5,6,7,8,13,21,22,23,24,25,26,27,28,29,30,31,32,33,34,35,36,37,38,39,40,41,42,43,44,45,46,47,48,49,50,51,52,53,54,55,56,57,58,59,60,61,62,63,64,65,66,67,68,69,70,71].

The characteristics of these studies are summarized in Table S3. There were twenty-eight cohort studies [6,7,8,13,24,29,32,33,34,35,37,43,44,48,50,54,56,57,58,59,60,61,62,65,66,67,68], twenty-one cross-sectional studies [21,27,28,30,31,36,38,40,41,42,45,47,49,51,52,53,63,64,69,70], three intervention studies [25,26,71], two meta-analyses [5,22], one case–control study [39], and one quasi-experimental study [23]. Most articles were published after 2015 (*n* = 41) and ~50% of them were published since 2020 (*n* = 21). Approximately 40% of the 54 observational or interventional studies were conducted in East and Southeast Asia (*n* = 23; Japan = 22, Malaysia = 1), followed by Europe (*n* = 18), Australia (*n* = 11), and Canada (*n* = 2). The sizes of these studies varied from 40 to 4487 patients. Most investigations did not focus on specific diseases or disabilities (*n* = 42), and several studies concerned patients after hip fractures (*n* = 7), strokes (*n* = 6), or spinal cord injuries (*n* = 1).

### 3.1. Measures for Identifying Malnutrition Used in the Literature

The eligible studies employed a variety of nutritional screening tools, assessment tools, and diagnostic criteria for malnutrition. Twenty-nine studies used malnutrition screening tools, among which twenty-six implemented the Mini Nutritional Assessment Short-Form (MNA-SF) [7,8,26,27,30,32,34,35,37,38,39,41,43,45,48,49,50,51,52,55,56,58,60,61,62]. Two studies used the Malnutrition Universal Screening Tool (MUST) [7,40], one study applied the Short Nutritional Assessment Questionnaire for Residential Care (SNAQ^RC^) [68], and one study used the Nutritional Risk Screening 2002 (NRS2002) [71]. Fifteen studies used nutritional assessment tools, of which ten used the MNA [5,13,24,25,27,31,42,47,57,70], five used the SGA [21,29,59,66,67], and one used the PG-SGA [13]. Regarding diagnostic criteria for malnutrition, twelve studies used the GLIM criteria [6,40,41,44,50,51,53,54,55,65,69], seven used the European Society for Clinical Nutrition and Metabolism (ESPEN) criteria [6,7,28,33,39,46,51], and two used the International Classification of Disease, Australian modification version (ICD-10-AM) [13,35]. Three studies used composite criteria of both body weight and weight loss, deemed nutritional screening tools [23,63,64,72].

Table 2 summarizes the components of measures for identifying malnutrition utilized in the selected studies. Except for body weight and weight change, which were inclusion criteria, the most common component was reduced nutrient intake (9/12 tools), followed by stress due to disease (6/12), physical function (4/12), cognitive function (2/12), and anthropometric indices (2/12). Other components included serum albumin, medication, dietary habits, comorbidities, age, and muscle and fat loss.

### 3.2. Prevalence of Malnutrition

The prevalence of malnutrition (and its risks) was reported in 41 studies. Among these investigations, seventeen used nutritional screening tools (thirteen utilized the MNA-SF [8,27,30,32,35,37,39,49,56,58,60,61,62], one used the Short Nutritional Assessment Questionnaire for Residential Care [SNAQ^RC^] [68], and three others utilized combined tools [25,63,64]). Twelve studies used nutritional assessment tools (seven utilized the MNA [5,13,24,27,31,47,70], four utilized the SGA [21,59,66,67], and one utilized the PG-SGA [13]), and twenty-two studies used diagnostic tools for malnutrition (twelve utilized GLIM criteria [6,28,40,41,44,50,51,53,55,65,69], seven utilized ESPEN criteria [6,7,33,39,46,51] and two studies, which analyzed identical datasets, utilized ICD-10-AM [13,35]). Malnutrition risk prevalence, evaluated by nutritional screening tools, varied widely (0.4–96.5%). Malnutrition prevalence using nutritional assessment tools was also widespread (MNA: 8–46.7%, SGA: 33.6–70.4%, PG-SGA: 52.6%). The proportion of malnutrition by ESPEN criteria was similar to that derived from nutritional assessment tools (12.6%–62.3%), whereas a higher prevalence was reported when applying GLIM criteria (29.0–82.1%).

### 3.3. Accuracy

Five studies examined accuracy. Three studies investigated the MNA-SF, with a sensitivity of 77–100% and a specificity of 23–85% [7,27,35]. Another two studies targeted nutritional assessment tools. An Australian study indicated the sensitivity and specificity of the MNA (57.7% and 96.8%) and PG-SGA (100% and 87.1%) for the ICD-10-AM [13]. Another study showed the correlation between the MNA and the Nutritional Form for the Elderly (NUFFE) (correlation coefficient: −0.74, *p* < 0.001) [57]. No studies examined the accuracy of diagnostic criteria for malnutrition.

### 3.4. Predictive Validity

**Figure 2:**
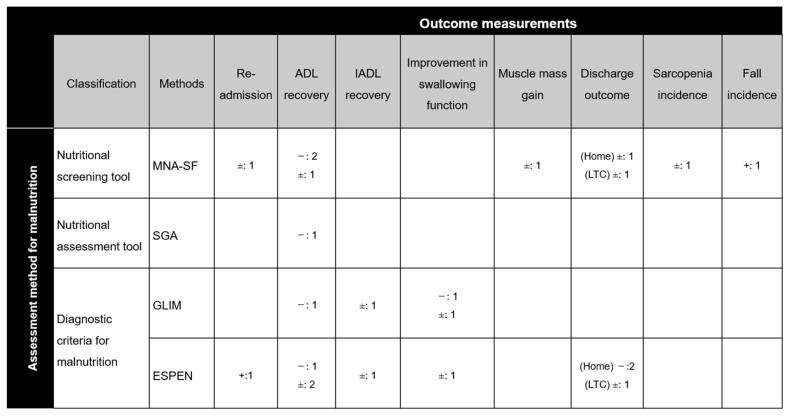
Mapping matrix for malnutrition measures and outcomes investigated in 12 cohort studies. ADL, activities of daily living; ESPEN, diagnostic criteria for malnutrition by the European Society for Parenteral and Enteral Nutrition; GLIM, diagnostic criteria for malnutrition by the Global Leadership Initiative on Malnutrition; IADL, instrumental activities of daily living; LTC, long-term care; MNA-SF, Mini Nutritional Assessment-Short Form; SGA, Subjective Global Assessment. “−”, “±”, and “+“ indicate a negative association, no association, and a positive association between measures for identifying malnutrition and outcomes, respectively. Values in each cell indicate the number of studies.

Many studies tested predictive validity, including mortality in two studies [29,61] re-admission to a hospital in two studies [29,35], physical performance in two studies [44,62], activities of daily living (ADL) in twelve studies [6,7,8,32,33,36,38,58,59,61,62,66], instrumental ADL (IADL) in one study [6], swallowing function in one study [39], body composition in one study [43], body weight in two studies [27,60], and other outcomes, e.g., oral health, complications, sarcopenia onset, tongue pressure, texture-modified diet, poor appetite, and falling, in thirteen studies [8,24,29,34,44,45,50,53,54,58,61,62,66] (Table S3). Figure 2 shows the mapping matrix of the predictive validity of nutritional assessment measures and outcome indicators from 12 cohort studies addressing confounding effects. ADL recovery was the most commonly used outcome measurement, followed by discharge outcomes, re-admission, IADL recovery, improvement in swallowing function, and other outcomes. The predictive validity of the MNA-SF for ADL recovery was inconsistent. This tool showed no association with other outcome measures other than falling [7,8,32,34,43,62]. Six studies investigated the predictive validity of diagnostic criteria for malnutrition (GLIM criteria [6,50,54] and ESPEN criteria [6,7,33,39]). Those results revealed some outcome measures that were related to diagnostic criteria and others that were not. In contrast, the predictive validity of nutritional assessment tools was rarely investigated, although one study examined the predictive validity of SGA for ADL recovery in patients with spinal cord injuries. It reported a negative correlation between malnutrition and functional gain [5].

## 4. Discussion

This comprehensive scoping review yielded three key findings in nutritional assessment for geriatric rehabilitation patients. First, a wide range of components, including functional assessments, were included in measures to identify malnutrition in geriatric rehabilitation patients. Second, few studies have examined the accuracy of malnutrition measures. Third, when limited to studies with appropriate design, few studies assessed predictive validity in this population.

### 4.1. Components of the Measures for Identifying Malnutrition

Since malnutrition is a multifactorial condition, the identified measures for malnutrition contained a variety of components that represent the phenotype, e.g., low BMI and weight loss, causes (reduced food intake and disease burden), and risk factors, e.g., decreased functioning and cognitive impairment (Table 2). Impaired physical functions, which are assessed by the MNA-SF and all nutritional assessment tools, may be causative factors for malnutrition in older adults with disabilities. However, this condition can also result from malnutrition [9]. The potential interaction between malnutrition and disabilities may increase the risk of overestimating malnutrition prevalence and predictive validity for functional outcomes. To overcome these issues, nutritional professionals should distinguish functional impairments related to malnutrition from those of non-nutritional origins, e.g., hemiparesis due to cerebrovascular disease [73].

### 4.2. Accuracy and Predictive Validity

Few studies have investigated the accuracy of measures to identify malnutrition in geriatric rehabilitation settings. Three of five eligible studies addressed nutritional screening tools. Although these are tools to identify individuals at risk of malnutrition, they do not assess nutritional status [74]. Regarding nutritional status, the PG-SGA had relatively high sensitivity and specificity, compared to the MNA in a small study (*n* = 57) [13]. More studies will be needed to clarify the accuracy of existing nutritional assessment tools with a sufficient number of participants. Furthermore, no study investigated the accuracy of diagnostic criteria for malnutrition in geriatric rehabilitation patients. Diagnostic frameworks, e.g., the GLIM criteria, the ESPEN criteria [75], and diagnostic characteristics of malnutrition from the American Society for Parenteral and Enteral Nutrition and the Academy of Nutrition and Dietetics [76], do not replace nutritional assessment tools, and they need to be validated against appropriate reference standards for malnutrition [77]. Given the scant evidence, future studies examining the accuracy of nutritional assessment tools and diagnostic criteria for malnutrition should use “semi-gold standard methods”, such as nutritional assessment tools or diagnoses of malnutrition, e.g., the International Classification of Disease [13,77], as a reference method.

The predictive validities of nutritional screening tools and diagnostic criteria in geriatric rehabilitation have been studied, although the results are divergent. Additionally, the predictive validity of nutritional assessment tools is lacking. We found one study using the SGA for ADL recovery in patients with spinal cord injuries [59]. Therefore, the predictive validities of nutritional assessment tools and diagnostic criteria for malnutrition in geriatric rehabilitation patients remain to be studied. A prospective cohort study applying a nutritional assessment tool or diagnostic criteria such as GLIM criteria should be conducted for functional recovery, a central measure in rehabilitation patients [78].

### 4.3. Usability and Considerations of Nutritional Assessment Tools in Geriatric Rehabilitation

The poor use of nutritional assessment tools in the literature suggests that comprehensive nutritional assessments are not commonly performed in routine clinical practice in geriatric rehabilitation. A Canadian study showed that 64% of dietitians do not use a validated nutritional assessment tool for patient recovery from stroke [79]. Additionally, an annual survey of convalescent rehabilitation wards in Japan in 2021 reported that only 5.4% of hospital wards routinely used nutritional assessment tools to identify malnutrition (unpublished data). Routine screening and subsequent assessment and diagnosis for malnutrition are highly encouraged for all older patients to identify those who may benefit from nutrition support, so as to maximize the patient’s functional recovery by providing individualized nutrition care [2]. Therefore, a well-designed, validated nutritional assessment tool should be used in clinical practice and for research in geriatric rehabilitation settings.

There are also other considerations when nutritional assessment tools are applied to geriatric rehabilitation patients. The MNA contains a “mobility” item with the options, “able to get out of bed/chair, but does not go out” or “goes out”. For older adults who need support for walking from caregivers due to hemiplegia or paraplegia, we may score them as this item, “able to get out of bed/chair, but does not go out”, because their mobility depends on the caregivers. Similarly, SGA involves the functional capacity with three options (working sub-optimally, ambulatory, and bedridden). Scoring this item for some patients in geriatric rehabilitation requires caution, because their functional limitation is due to disease, e.g., stroke or hip fracture, but not malnutrition [73]. The PG-SGA includes cancer-related symptoms such as nausea, vomiting, and smell/ taste change, and some options are also suitable for older rehabilitation patients. Questions about activities and function over the preceding month may be biased against patients who stayed at an acute care hospital during this period [12]. In addition to detailed, standard nutritional assessment, rehabilitation goals and programs should be involved in a comprehensive evaluation. This information is important for setting energy requirements because activity-related energy expenditure in geriatric rehabilitation patients seems more attributable to total energy expenditure than it is for acute care patients.

### 4.4. Application of the Global Leadership Initiative on Malnutrition Criteria in Geriatric Rehabilitation

The application of GLIM criteria is an emerging issue in geriatric rehabilitation settings [78], because identifying malnutrition and providing appropriate nutritional care is essential for malnourished patients with disabilities. However, criteria have not been well validated for these patients to date. The clinical application of the GLIM criteria may be suboptimal in in-patient rehabilitation. For example, approximately 6% of convalescent rehabilitation wards in Japan employ it to identify malnutrition, as reported in the 2021 annual survey (unpublished data). Although some barriers to the use of GLIM criteria in rehabilitation medicine, e.g., the correct measurement of muscle mass for people with disabilities, still exist [78], applying common criteria for malnutrition would encourage the exploration of the true effect of malnutrition in different geographical locations and the improvement of nutritional care for geriatric rehabilitation patients.

### 4.5. Potential Biases in the Review Process

There are several risks of bias in this review. First, since a global definition of a “nutritional assessment tool” is lacking, we used the Academy of Nutrition and Dietetics criteria. Accordingly, the literature search involved some nutritional screening tools such as the MNA-SF, but others such as the Malnutrition Screening Tool [35] and the NUFFE [57], which are often used and recommended for older patients in rehabilitation settings, were excluded. We do not regard this as a significant flaw in the current review because it focused on measures for identifying malnutrition, not on screening tools. Second, we did not strictly define geriatric rehabilitation settings when selecting search terms because healthcare systems that provide rehabilitation for older patients are rather heterogeneous; thus, study populations may vary widely [80,81,82]. Moreover, the length of rehabilitation also varied widely among included studies. As a result, the predictive validities of the measures used in different studies may not be comparable. The results of this review, therefore, should be interpreted with caution.

### 4.6. Agreements and Disagreements with Other Studies or Reviews

This review is the first study focusing on nutritional assessment in geriatric rehabilitation patients. Power et al. comprehensively reviewed 119 validation studies for nutritional screening and found that 34 tools had been validated. They concluded that the NUFFE was the most useful tool for rehabilitation patients [74]. However, nutritional assessment was not the purpose of that review; hence, its results are not comparable to those presented here. Moloney et al. published a scoping review for nutritional assessment and intervention to prevent and treat malnutrition in older people living in community and long-term care facilities [18]. They found a sufficient number of validation studies of nutritional assessment tools to conduct a systematic review in this setting. Based on that review, the Academy of Nutrition and Dietetics published the Malnutrition in Older Adults Evidence-Based Nutrition Practice Guidelines [83]. These guidelines recommend the MNA for older adults in long-term care and community. Additionally, the guidelines indicate that SGA and PG-SGA can be alternatives for seniors in long-term care if the MNA is not feasible, while SGA may also be useful in community settings. In contrast, we found only one validation study for geriatric rehabilitation. However, these results are unlikely to conflict, because the Academy’s recommendation targeted older adults in community and long-term care.

## 5. Conclusions

This scoping review produced three key findings for measures identifying malnutrition in geriatric rehabilitation: (1) components of malnutrition measures involved phenotypes, causes, and risk factors for malnutrition, including physical and cognitive function. This characteristic may risk the overestimation of malnutrition prevalence and predictive validity. (2) Few studies have investigated the accuracies of nutritional assessment tools, and no study has described malnutrition diagnostic criteria. (3) The predictive validities of measures to identify malnutrition were inconsistently reported and the evidence for nutritional assessment tools is particularly scarce. The operational definition of nutritional assessment and geriatric rehabilitation might be the limitation of this review. Further study will be required to overcome these issues in order to establish optimal nutrition assessment and diagnosis of malnutrition to provide the best nutritional care for geriatric rehabilitation patients.

## Figures and Tables

**Table 1:** Patient, concept, and context framework utilized in this scoping review.

Items	Contents
Patient	Older patients in geriatric rehabilitation settings with a mean age of 65 or older
Concept	Types and characteristics of nutritional assessment methods, accuracy, and outcome measures related to malnutrition, identified by these tools or to the single parameter used in these tools
Context	Any study that utilized nutritional assessment methods for identifying malnutrition in older adults admitted to rehabilitation hospital/ward/facility or equivalent setting. Descriptive studies or studies that used nutritional assessment but focused on other variables were not excluded.
Design	Cross-sectional, cohort, case–control, systematic reviews, meta-analyses, and intervention studies.

**Table 2:** Components of tools used for nutritional assessment in selected publications.

Category	Methods	Body Weight/BMI	Weight Loss	Appetite Loss/Reduced Nutritional Intake	Anthropo-Metric Indices	Physical Functions/Activities	Cognitive Functions	Stress/Inflammatory Response from Disease	Others
Nutritional screening tool	MNA-SF	✔	✔	✔	(✔) ^a^	✔	✔	✔	
	MUST	✔	✔	✔				✔	
	NRS2002	✔	✔	✔				✔	✔ ^b^
	SNAQ^RC^	✔	✔	✔					✔ ^c^
	Laporte’s method [23,68]	✔	✔						✔ ^d^
	van Zwienen-Pot’s method [63,64]	✔	✔						
Nutritional assessment tool	MNA	✔	✔	✔	✔	✔	✔	✔	✔ ^e^
	SGA	✔	✔	✔		✔		✔	
	PG-SGA	✔	✔	✔		✔			✔ ^f^
Diagnostic criteria for malnutrition	GLIM	✔	✔	✔				✔	✔ ^g^
	ESPEN	✔	✔						✔ ^h^
	ICD-10-AM	✔	✔	✔					✔ ^i^

ESPEN, the European Society for Clinical Nutrition and Metabolism; ICD-10-AM, the International Statistical Classification of Diseases and Health Related Problems, 10th revision, Australian Modification; GLIM, the Global Leadership Initiative for Malnutrition; MNA, Mini Nutritional Assessment; MNA-SF, Mini Nutritional Assessment Short Form; MUST, Malnutrition Universal Screening Tool; NRS2002, Nutritional Risk Screening 2002; PG-SGA, Patient-Generated Subjective Global Assessment; SGA, Subjective Global Assessment; SNAQ^RC^, Short Nutritional Assessment Questionnaire for Residential Care. ^a^ Calf circumference measurement if BMI is unavailable. ^b^ Age. ^c^ Capability of eating/drinking. ^d^ Serum albumin. ^e^ Residency, medication, pain/ulcer, dietary and fluid intake, needs for assistant in eating, self-perceived nutritional status and health status, diet type, GI symptoms, muscle loss, fat loss, edema/ascites. ^f^ Symptoms related to appetite loss, comorbidities, fever, steroid use, muscle loss, fat loss, edema/ascites. ^g^ Muscle loss, reduced assimilation. ^h^ Fat-free mass loss. ^i^ Muscle loss, fat loss.

## Supplementary Materials

The following supporting information can be downloaded at: https://www.mdpi.com/article/10.3390/nu16020223/s1, Table S1: inclusion and exclusion criteria for the literature search. Table S2: search terms for MEDLINE and CINAHL. Table S3: summary of the 56 studies included in this scoping review.

## Abbreviations

ADL: activities of daily living; BMI: body mass index; ESPEN, the European Society for Clinical Nutrition and Metabolism; IADL, instrumental activities of daily living; ICD-10-AM, the International Statistical Classification of Diseases and Health Related Problems, 10th revision, Australian Modification; GLIM, the Global Leadership Initiative for Malnutrition; MNA, Mini Nutritional Assessment; MNA-SF, Mini Nutritional Assessment Short Form; MUST, Malnutrition Universal Screening Tool; NRS2002, Nutritional Risk Screening 2002; NUFFE, Nutrition Form For the Elderly; PG-SGA, Patient-Generated Subjective Global Assessment; SGA; Subjective Global Assessment; SNAQ^RC^, Short Nutritional Assessment Questionnaire for Residential Care.
