# Supplementary material for: Measures for Identifying Malnutrition in Geriatric Rehabilitation: A Scoping Review
Source: Nutrients. 2024 Jan 10;16(2):223. doi: 10.3390/nu16020223 (PMC10820477; doi:10.3390/nu16020223)
Supplement: Supplementary file 1 [file nutrients-16-00223-s001.zip › nutrients-2794801-supplementary.pdf]

Table S1. Inclusion and exclusion criteria for the literature search.

	Inclusion criteria	Exclusion criteria
Language	English	Non-English
Publication date	From January 1, 1980 to September 30 2023	Before 1980
Population	Older adults (mean age 65 years or over) undergoing rehabilitation	Individuals aged less than 65 years, no rehabilitation provided
Setting	Rehabilitation hospital, rehabilitation unit/ ward, rehabilitation facility, post-acute care unit/ward, convalescent rehab ward	Acute care hospital, long-term care hospital, long-term care facility, community
Definition of nutritional assessment	Containing both static measurement of body weight/ body composition and body weight change	Not containing either static measurement of body weight/ body composition and body weight change
Study design	Cross-sectional study, cohort study, case-control study<br>intervention study (only used to look for nutritional assessment tools used)<br>The studies in systematic reviews and meta-analysis were also included.	Conference abstracts, conference papers, qualitative studies, study protocols, commentaries, editorials, and narrative reviews.

Table S2. Search terms for MEDLINE and CINAHL

MEDLINE		
	Search terms	Hits
#1	exp Malnutrition/ OR (malnutrition OR malnourished OR undernutrition OR undernourished OR "under nutrition" OR "decreased nutritional status" OR "nutritional disorder" OR "nutritional deterioration" OR "protein energy malnutrition" OR "protein calorie malnutrition").mp.	178031
#2	("rehabilitation unit*" OR "rehabilitation ward*" OR "rehabilitation hospital*" OR "geriatric ward*" OR "post-acute" OR "rehabilitation facilit*" OR "geriatric facilit*" OR "geriatric rehabilitation").mp.	20101
#3	#1 AND #2	418
#4	limit 3 to (english language and yr="1980 - 2023" and "all aged (65 and over)")	245
CINAHL		
	Search terms	Hits
#1	(MH Malnutrition+) OR (malnutrition OR malnourished OR undernutrition OR undernourished OR "under nutrition" OR "decreased nutritional status" OR "nutritional disorder" OR "nutritional deterioration" OR "protein energy malnutrition" OR "protein calorie malnutrition")	27722
#2	("rehabilitation unit*" OR "rehabilitation ward*" OR "rehabilitation hospital*" OR "geriatric ward*" OR "post-acute" OR "rehabilitation facilit*" OR "geriatric facilit*" OR "geriatric rehabilitation")	14327
#3	S1 AND S2	200
#4	Limiters - Published Date: 19800101-20230930; English Language; Age Groups: Aged: 65+ years	117

Table S3. Summary of 56 studies included in this scoping review

1st author, publication year	Country	N	Design	Measures for identifying malnutrition[a]	Concurrent validity	Outcome measures for predictive validity	Potential confounders adjusted in statistical analysis
Andersson, 2002 (21)	Sweden	223	Cross-sectional	SGA	-	Oral health problems (e.g., voice, lips)	-
Avenell, 2016 (22)	Diverse	3881/ 41 studies	MA	Unspecified	-	-	-
Babineau, 2008 (23)	Canada	62	Quasi-experimental study	(1) BMI and serum albumin, or (2) BMI and weight loss	-	-	-
Bellelli, 2012 (24)	Italy	502	Cohort	MNA	-	Complications	-
Bouillanne, 2013 (25)	France	66	Intervention	MNA	-	-	-
Bouillanne, 2014 (26)	France	66	Intervention	MNA-SF	-	-	-
Charlton, 2010 (27)	Australia	2076	Cross-sectional	MNA, MNA-SF, MNA-SFver2	(MNA-SF) Se:89% Sp: 44%	Body weight	-

Clark, 2020 (28)	Australia	444	Cross-sectional	GLIM, ESPEN	-	-	-
Duerksen, 2000 (29)	Canada	87	Cohort	SGA	-	Mortality<br>Re-hospitalization<br>Length of institutionalization	-
Frangos, 2016 (30)	Switzerland	392	Cross-sectional	MNA-SF	-	-	-
Groenendijk, 2020 (31)	Netherlands	40	Cross-sectional	MNA	-	-	-
Hettiarachchi, 2021 (6)	Australia	618	Cohort	GLIM, ESPEN	-	ADL<br>IADL	Age, CCI, cognitive impairment
Lelli, 2019 (32)	Italy, Spain	415	Cohort	MNA-SF	-	ADL	Age, sex, MMSE, CCI, LOS, BI at admission, diagnosis
Maeda, 2018 (33)	Japan	207	Cohort	ESPEN	-	ADL	Age, sex, LOS in the acute ward, CCI, premorbid BI, LOS in the post-acute care ward
Malafarina, 2019 (34)	Spain	187	Cohort	MNA-SF	-	Sarcopenia	Age, sex, research cites

Marshall, 2016a (13)	Australia	57	Cohort	MNA, scored PG-SGA, ICD-10-Australian modification	(MNA)<br>Se: 58%<br>Sp: 97%<br>(PG-SGA rating))<br>Se: 100%<br>Sp: 87%<br>(PG-SGA score)<br>Se: 92%<br>Sp: 84%	LOS<br>Admission to RACF<br>Discharging to home	-
Marshall, 2016b (35)	Australia	57	Cohort	MNA-SF, ICD-10-Australian modification	(MNA-SF)<br>Se: 100%<br>Sp: 23%	Re-hospitalization	-
Nishioka, 2016a (36)	Japan	178	Cross-sectional	MNA-SF	-	ADL	Age, sex, LOS, days from onset to admission, FIM on admission, lower-order items of the MNA-SF
Nishioka, 2016b (37)	Japan	897	Cohort	MNA-SF	-	-	-
Nishioka, 2018 (38)	Japan	110	Cross-sectional	MNA-SF	-	ADL	Age, sex, pre-stroke certification for LTCI,

							FIM admission, CCI, days from fracture to admission
Nishioka, 2020a (7)	Japan	420	Cohort	ESPEN, MUST, MNA-SF	(MNA-SF) Se: 78-96% Sp: 57-85%	ADL Discharge to home Discharge to LTCF Re-admission (The effect of the MUST was not analyzed)	Age, sex, onset-admission duration, stroke type, dysphagia, dyslipidemia, pre-stroke ADL, FIM
Nishioka, 2020b (39)	Japan	113	Case-control	ESPEN, MNA-SF	-	Swallowing ability (The effect of the MNA-SF was not analyzed)	Age, gender, FILS on admission, FIM on admission
Nishioka, 2021a (8)	Japan	489	Cohort	MNA-SF	-	ADL Discharging to home	Age, sex, CCI, onset admission duration, need of care before onset, FILS, daily rehabilitation dose

Nishioka, 2021b (40)	Japan	531	Cross-sectional	GLIM, MUST	-	-	-
Nomoto, 2022 (41)	Japan	160	Cross-sectional	GLIM, MNA-SF	-	-	-
O'Leary, 2011 (42)	Australia	52	Cross-sectional	MNA	-	LOS	Age, sex
Ogawa, 2022 (43)	Japan	807	Cohort/ Cross-sectional	MNA-SF	-	Body composition	Age, sex, BMI on admission, SMI on admission, CCI, length from onset to transfer to the hospital, fracture types, length of hospital stay, period of exercise therapy
Rojer, 2021 (44)	Australia	145	Cohort	GLIM	-	Physical performance Sedentary behavior	Age, sex, comorbidity
Sakai, 2017 (45)	Japan	174	Cross-sectional	MNA-SF	-	Tongue pressure	Age, complete denture, BI, serum albumin, CONUT score, BMI, handgrip strength, CC,

							arm muscle area.
Sánchez-Rodríguez, 2017 (46)	Spain	88	Cohort	ESPEN	-	-	-
Savina, 2003 (47)	Italy	103	Cross-sectional	MNA	-	-	-
Shimazu, 2021 (48)	Japan	454	Cohort	MNA-SF	-	-	-
Shimizu, 2018 (49)	Japan	188	Cross-sectional	MNA-SF	-	Texture-modified diet	Age, sex, primary disease, SMI, FIM
Shimizu, 2019 (50)	Japan	188	Cohort	GLIM, MNA-SF	-	Texture-modified diet (The effect of the MNA-SF was not analyzed)	Age, sex, IDDSI-FDS, LOS, history of stroke, type of stroke, CCI, OHAT, FIM
Shimizu, 2020 (51)	Japan	335	Cross-sectional	GLIM, ESPEN, MNA-SF	-	-	-
Shimizu, 2021a (52)	Japan	129	Cross-sectional	MNA-SF	-	-	-

Shimizu, 2021b (53)	Japan	208	Cross-sectional	GLIM	-	Poor appetite	Age, sex, primary disease, days from stroke onset to rehabilitation, CCI, MMSE, FIM
Shimizu, 2021c (54)	Japan	146	Cohort/ Cross-sectional	GLIM	-	Swallowing ability Sarcopenic dysphagia	Age, sex, primary disease, CCI, MMSE, FIM, FILS, probable sarcopenic dysphagia
Shimizu, 2021d (55)	Japan	380	Cross-sectional	GLIM, MNA-SF	-	-	-
Shiraishi, 2017 (56)	Japan	108	Cohort	MNA-SF	-	-	-
Söderhamn, 2002 (57)	Sweden	114	Cohort	MNA	(MNA) r=-0.74 (P<0.001)	-	-
Tamamura, 2021 (58)	Japan	155	Cohort	MNA-SF	-	ADL Discharging to home	-
Tanaka, 2019 (59)	Japan	154	Cohort	SGA	-	ADL	Age, sex, ASIA, FIM
Than, 2019 (60)	Australia	4487	Cohort	MNA-SF	-	Body weight	-

Thiam, 2022 (61)	Malaysia	73	Cohort	MNA-SF	-	Mortality<br>ADL<br>Complications	-
Urquiza, 2020 (62)	Spain	375	Cohort	MNA-SF	-	Physical performance<br>ADL<br>Falling	Age-adjusted CCI, MMSE, SPPB, POMA, FAC (adjusted only for falling)
van Zwienen-Pot, 2017 (63)	Netherlands	245	Cross-sectional	Combined methods with weight loss and BMI	-	-	-
van Zwienen-Pot, 2018 (64)	Netherlands	79	Cross-sectional	Combined methods with weight loss and BMI	-	-	-
Verstraeten, 2021 (65)	Australia	506	Cohort	GLIM	-	-	-
Vivanti, 2011 (66)	Australia	194	Cohort	SGA	-	ADL<br>LOS<br>Falling	-
Wester, 2018 (67)	Australia	100	Cohort	SGA	-	-	-
Wojzischke, 2020 (5)	Multi-country	19127/62	MA	MNA	-	ADL	-

		studies					
Nijmeijer, 2023 (68)	Netherland	291	Cohort	$SNAQ^{RC}$	-	-	-
Blanquet, 2023 (69)	France	360	Cross-sectional	GLIM	-	Intake-requirement gap for energy and protein	-
Kayhan Kocak, 2023 (70)	Turkey	585	Cross-sectional	MNA	-	Re-hospitalization	-
Kurmann, 2023 (71)	Switzerland	203	Intervention	NRS2002	-	-	-

ADL, activities of daily living; ASIA, American Spinal Injury Association; Impairment Scale; BI, Barthel Index; BMI, body mass index; BNP, brain natriuretic peptide; CC, calf circumference; CCI, Charlson Comorbidity Index; CONUT, CONtrolling NUTritional status; ESPEN, diagnostic criteria for malnutrition by the European Society for Clinical Nutrition and Metabolism; FAC, Functional Ambulation Category; FILS Food Intake LEVEL Scale; FIM, Functional Independence Measure; GLIM, Global Leadership Initiative for Malnutrition; GNRI, Geriatric Nutritional Risk Index; IADL, instrumental activities of daily living; ICD-10-AM, International Classification of Disease, 10th version, Australian modification; IDDSI-FDS, International Dysphagia Diet Standardization Initiative Functional Diet Scale; LOS, length of stay; LTCF, long-term care facilities; LTCI, long-term care insurance; MA, meta-analysis; MMSE, Mini Mental State Examination; MNA-SF, Mini Nutritional Assessment- Short Form; MNA, Mini Nutritional Assessment; MUST, Malnutrition Universal Screening Tool; NRS2002, Nutritional Risk Screening 2002; OHAT, Oral Health Assessment Tool; PG-SGA, patient-generated Subjective Global Assessment; POMA, Tinetti Performance-Oriented Mobility Assessment; RACF, residential aged-care facilities; Se, sensitivity; Sp, specificity; SGA, Subjective Global Assessment; SMI, skeletal muscle mass index; $SNAQ^{RC}$, Short Nutritional Assessment Questionnaire for Residential Care; SPPB, Short Physical Performance Battery. [a]Measures for malnutrition not meeting pre-specified criteria (including both static body weight and weight change) were omitted from this table (e.g., Malnutrition Screening Tool)
